# Supplementary material for: Empirical methods for controlling false positives and estimating confidence in ChIP-Seq peaks
Source: BMC Bioinformatics. 2008 Dec 5;9:523. doi: 10.1186/1471-2105-9-523 (PMC2628906; doi:10.1186/1471-2105-9-523)
Supplement: Additional File 1 — A variety of html documents from the USeq web site detailing the available applications, their best usage, output file type descriptions, command line menus, etc. [file 1471-2105-9-523-S1.zip › USeqUserGuides/JavaDoc.zip!/JavaDoc/trans/anno/package-summary.html]

trans.anno


|  |  |  |
| --- | --- | --- |
| **Overview** \| **Package** \| Class \| **Use** \| **Tree** \| **Deprecated** \| **Index** \| **Help** | |  |
| **PREV PACKAGE**   **NEXT PACKAGE** | **FRAMES**    **NO FRAMES**     **All Classes** |  |


---

## Package trans.anno

For associating intervals, binding regions, and binding peaks with GFF annotation.

**See:**
  
          **Description**

| **Class Summary** | |
| --- | --- |
| **AnnotateRegions** | For annotating Intervals or binding regions with gene information, proximity, neighbors, and bias based on random trials. |
| **AnnotateRegionsWithGeneList** | For annotating a picks file (chr, start, stop) with gene information for a particular list of CG names. |
| **BindingRegion** | For holding information about a genomic interval, what genes are up, down, overlap, or contain this genomic inteval. |
| **BindingRegionComparator** |  |
| **BindingRegionsToGff3** | Converts a Binding GenomicRegion file into GFF3 format. |
| **BindingRegionsToIntervals** | Converts binding regions to intervals setting the binding region as the best window and adds 2kb on either side. |
| **DmelCGNameCoordinatePrinter** | Prints genome coordinates for Dmel Rel 4 GFF3 CG names. |
| **FindNeighboringGenes** | Finds closest genes and all genes within a given neighborhood. |
| **Gff2ToBindingRegions** | Converts a gff2 file to a binding region file (chr start end mockSequence). |
| **Gff2ToIntervals** | Converts a gff2 file to an interval object file. |
| **IntersectBindingPeaks** | Performs an intersection analysis on two peak pick files (chr peakPosition score). |
| **IntersectKeyWithRegions** | Takes a key from a spike in experiment and sorted lists of regions to calculate TPR, FPR, and FDR for every threshold |
| **IntersectRegions** | Performs an intersection analysis on binding region files (chr start stop (optional fraction gc)). |
| **MergeRegions** | Takes several tab delimited region files (chrom, start, stop, ...), creates the union and writes to disk. |
| **RandomRegions** | Use to generate random regions of a defined length from a file of regions (chrom, start, stop). |
| **Region** | Represents a genomic region. |
| **RegionComparator** |  |
| **SubtractRegions** | Intersects two lists of regions subtracting the first from the second. |

## Package trans.anno Description

For associating intervals, binding regions, and binding peaks with GFF annotation.

---


|  |  |  |
| --- | --- | --- |
| **Overview** \| **Package** \| Class \| **Use** \| **Tree** \| **Deprecated** \| **Index** \| **Help** | |  |
| **PREV PACKAGE**   **NEXT PACKAGE** | **FRAMES**    **NO FRAMES**     **All Classes** |  |


---
